# Attraction of *Lutzomyia longipalpis* to synthetic sex-aggregation pheromone: Effect of release rate and proximity of adjacent pheromone sources

**DOI:** 10.1371/journal.pntd.0007007

**Published:** 2018-12-19

**Authors:** Melissa J. Bell, Luigi Sedda, Mikel A. Gonzalez, Cristian F. de Souza, Erin Dilger, Reginaldo P. Brazil, Orin Courtenay, James G. C. Hamilton

**Affiliations:** 1 Division of Biomedical and Life Sciences, Faculty of Health and Medicine, Lancaster University, Lancashire, United Kingdom; 2 Centre for Health Informatics Computation and Statistics (CHICAS), Lancaster Medical School, Faculty of Health and Medicine, Lancaster University, Lancashire, United Kingdom; 3 Laboratório de Doenças Parasitárias, Instituto Oswaldo Cruz, FIOCRUZ, Rio de Janeiro, Brazil; 4 School of Life Sciences, University of Warwick, Coventry, United Kingdom; University of Notre Dame, UNITED STATES

## Abstract

In South America, the Protist parasite that causes visceral leishmaniasis, a potentially fatal human disease, is transmitted by blood-feeding female *Lutzomyia longipalpis* sand flies. A synthetic copy of the male produced sex-aggregation pheromone offers new opportunities for vector control applications. We have previously shown that the pheromone placed in plastic sachets (lures) can attract both females and males to insecticide treated sites for up to 3 months. To use the pheromone lure in a control program we need to understand how the application of lures in the field can be optimised. In this study we investigated the effect of increasing the number of lures and their proximity to each other on their ability to attract *Lu*. *longipalpis*. Also for the first time we applied a Bayesian log-linear model rather than a classic simple (deterministic) log-linear model to fully exploit the field-collected data. We found that sand fly response to pheromone is significantly related to the quantity of pheromone and is not influenced by the proximity of other pheromone sources. Thus sand flies are attracted to the pheromone source at a non-linear rate determined by the amount of pheromone being released. This rate is independent of the proximity of other pheromone releasing traps and indicates the role of the pheromone in aggregation formation. These results have important implications for optimisation of the pheromone as a vector control tool and indicate that multiple lures placed in relatively close proximity to each other (5 m apart) are unlikely to interfere with one another.

## Author summary

*Lutzomyia longipalpis* sand flies are the insect vectors of the Protist parasite *Leishmania infantum* which causes visceral leishmaniasis (VL) in Brazil. Control of VL has focussed on vector and infected reservoir control, but despite the sustained efforts of the Brazilian Health authorities the disease burden doubled between 1990 to 2016. New approaches to VL control are urgently needed. We previously demonstrated that *Lu*. *longipalpis* synthetic sex-aggregation pheromone placed alongside insecticide sprayed surfaces can attract and kill female sand flies. However, before the synthetic pheromone can be effectively exploited in any VL control program it is essential to understand how it might be deployed. In this study we investigated the effect of different amounts of pheromone and the spatial relationship between different pheromone sources on *Lu*. *longipalpis* catches. We developed a robust Bayesian analysis to fully exploit the field data which showed that optimal use of the pheromone could be achieved by placing individual or small numbers of pheromone releasing devices (lures) within the peridomestic environment and these can be positioned relatively closely without competing with each other. The results also revealed the significance of the pheromone in maintaining aggregations of *Lu*. *longipalpis* and suggested that *Lu*. *longipalpis* may be more evenly distributed in the peridomestic environment than previously recognised.

## Introduction

The sand fly *Lutzomyia longipalpis* (Diptera: Psychododae) is the major vector of *Leishmania infantum*, a Protist parasite and the causative agent of Zoonotic Visceral Leishmaniasis (ZVL) in Latin America. Approximately 90% of the cases of ZVL that occur in the Americas are recorded in Brazil where the greatest number of cases were found in the North East of the country [1]. The range of the vector has been gradually expanding and consequently human and canine cases of the disease are now found throughout the central and southern states where it was previously absent [1, 2].

In Minas Gerais State, Brazil, ZVL transmission is intense and over the last few decades has expanded from rural regions into cities [3, 4]. Incidence rates within the state were 1.6 per 100,000 inhabitants in 2012 and 1.4 per 100,000 in 2013, almost equal with incidence rates in North-eastern Brazil [5]. The causes of ZVL urbanisation are unclear but it is likely to be related to the movement of people and their animals from rural to urban settings as well as the ability of the vector to adapt to an urban environment [1, 6].

In Governador Valadares (GV) (a municipality in eastern Minas Gerais State) and the surrounding areas, ZVL transmission was believed to have been reduced after intervention with DDT spraying and extensive dog culling in the 1960’s [7]. However, it has re-emerged as a public health concern after the control campaign was interrupted in the 1990’s [8, 9]. From 2008 until 2013, 127 human cases were recorded with a fatality rate of 16%. In 2015–2017 the Centre for Control of Zoonoses (CCZ) reported 53 cases of human VL with 9 deaths [10].

Domestic dogs infected with *Le*. *infantum* are the proven reservoir host of human infection [1, 11]. Studies in GV in 2007 found that an average of 13% of dogs from 175 samples obtained across 2 districts; one urban and one rural, were seropositive [8]. That average had risen to 30.2% during 2008–2011 from 16,529 dog samples taken from 35 urban and rural districts of GV [9].

Leishmaniasis control relies on control of the sand fly vector via 1./ reactive spraying of insecticide as recommended by the Ministry of Health (MoH) 2./ reservoir control which is focused on the proactive diagnosis and removal of infected dogs and 3./ the use of therapeutic drugs [12]. However, despite these intensive efforts the vector and disease continue to affect new areas of the country [1].

*Lu*. *longipalpis* is a species complex and there are divergent views on how to define the members of the complex in Brazil. However, they can be distinguished from each other by the sex-aggregation pheromone that is produced by the males of each of the members of the complex [13]. Males of the most widespread member of the complex in Brazil, and the rest of South and Central America, produce (*S*)-9-methylgermacrene-B (9MGB) [13]. The *Lu*. *longipalpis* sex-aggregation pheromone attracts both males and females to mating aggregations (leks) which can become very large and well established in a particular location, with males returning night after night to compete with each other for access to females [14].

A synthetic copy of the 9MGB pheromone has been developed and experiments with a prototype lure, which allowed sustained controlled release of the pheromone for 24 hours [15], showed that increasing the quantity of prototype lures (which is equivalent to increasing the amount of pheromone being released and therefore equivalent to a greater number of males) from 1 to 10 increased the numbers of female *Lu*. *longipalpis* attracted by 278% [16]. Subsequently a long-lasting version of the lure which was attractive to females and males for up to 3 months in the field was developed. The long-lasting lure releases synthetic pheromone at an average rate (which is likely to be influenced by the ambient temperature) of approximately 4–7μg h^-1^. This is equivalent to the estimated range of pheromone produced by a natural lek (1μg h^-1^ to over 10μg h^-1^) [15, 18]. Located inside or next to pyrethroid insecticide-sprayed chicken sheds, the pheromone could be used as the attractive element of a ‘lure-and-kill’ (sometimes referred to as “attract-and-kill”) strategy for vector control.

In the current study we investigated the potential to improve sand fly capture rates by increasing the numbers of long-lasting lures at the trapping site (equivalent to increasing the release rate of pheromone). We also wished to establish the size of area around the long-lasting lure that might be under the influence of the pheromone as this has i) direct implications for the spatial deployment of an “attract-and-kill” intervention, and ii) may enhance our understanding of sand fly dynamics under experimental conditions. For example, in previous experiments with synthetic pheromone we established an experimental protocol whereby sand fly catches in pheromone baited traps were compared with those of un-baited control traps in a variety of situations [15–18]. However, the extent to which the catches of the control traps are influenced by the proximity of the pheromone baited traps is unknown. Based on limited laboratory-based wind-tunnel experiments pheromone (with host odour present) appears to attract females over 2.4m [19], thus control traps have typically been placed 3m from test traps. From a control perspective, it is important to understand the likely influence of adjacent “lure-and-kill” focal points on each other, in order to optimise their placement. Therefore, we tested the potential interaction between adjacent pheromone baited traps by increasing the distance between them.

## Materials and methods

### Study area

The study was carried out in Governador Valadares (Minas Gerais State, Brazil), a city of approximately 280,000 people 320 km northeast of Belo Horizonte the state capital. The city is located in the valley of the River Doce where, according to the Köppen—Geiger classification, the climate is temperate, characterised by dry winters and hot wet summers [20]. High densities of 9MGB sex-aggregation pheromone producing *Lu*. *longipalpis* have been found in several districts of Governador Valadares where *Lu*. *intermedia* and *Lu*. *cortelezzii*, vectors of American cutaneous leishmaniasis (ACL), have also been found [8, 9].

### Sampling sites and study design

**Fig 1:**
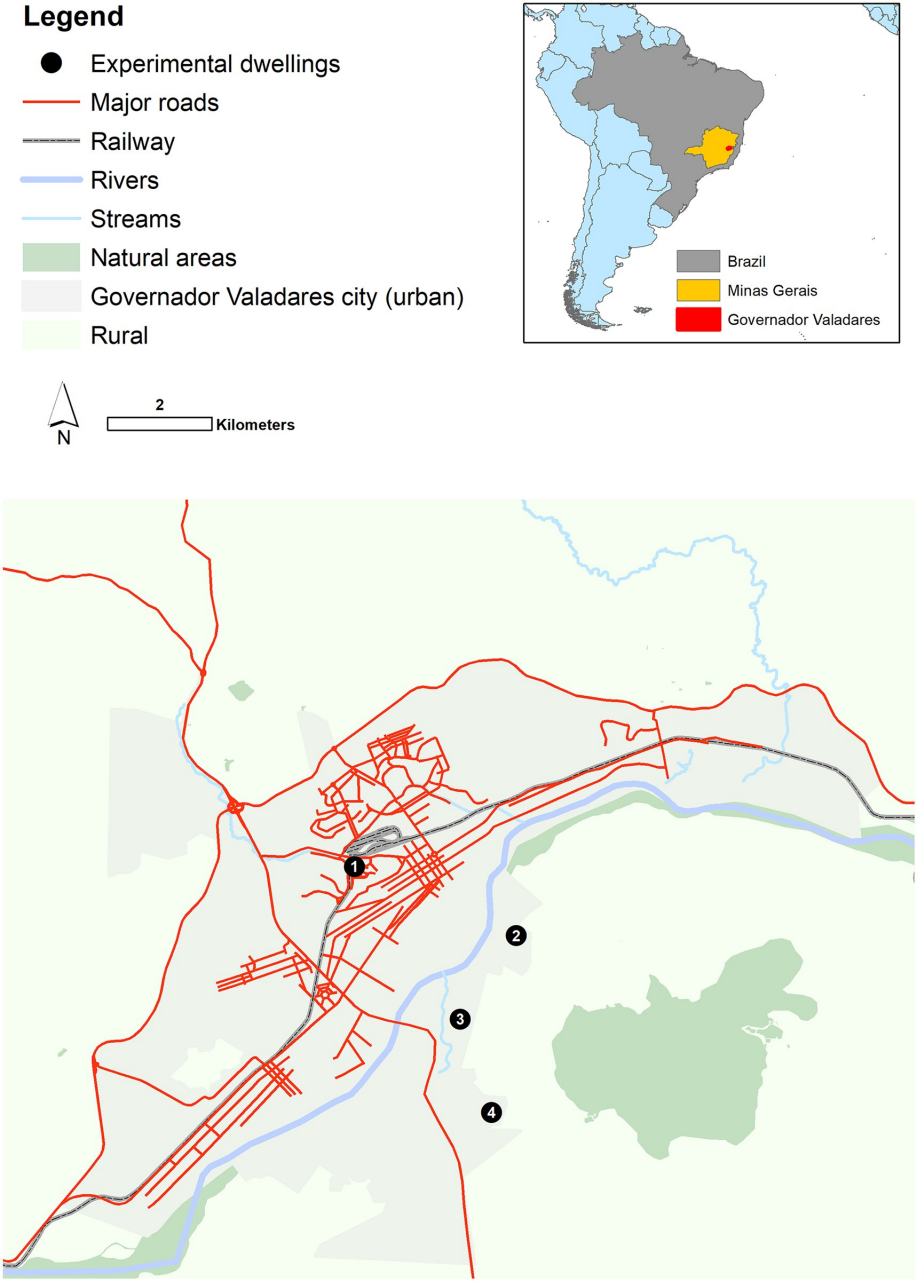
Location of the study site in Governador Valadares, Minas Gerais, Brazil. *The four study houses*, *in separate neighbourhoods are indicated by the black solid circles; 1—Vila Mariana; 2—Village da Serra; 3—Vila Parque Ibituruna; 4—Chacaras Recanto da Cachoeira*. [Map produced in ArcGIS 10.4.1, Base layers main map: OpenStreetMap (https://www.openstreetmap.org/search?query=Governador%20Valadares#map=13/-18.8593/-41.9381); Base layers inset map: ESRI World Countries Layer (https://www.arcgis.com/home/item.html?id=ac80670eb213440ea5899bbf92a04998)].

Experiments were conducted in 4 houses, based within four separate neighbourhoods of the city (Fig 1).

House 1 was in Vila Mariana within the urban perimeter of the city, not far from the city centre, whilst the other 3 houses were on the opposite side of the Rio Doce and outside the main urban perimeter. House 3 in Vila Parque Ibituruna was considered semi urban and the two houses in Village da Serra (House 2) and Chacaras Recanto da Cachoeria (House 4) were considered to be rural properties. The inclusion criteria for households were; accessibility, the ownership of chickens (>5) and *Lu*. *longipalpis* catch rate (average >15, males and females combined per trap per night) and finally the home owners’ willingness to comply with the long-term study requirements. A pre-experimental investigation in 20 houses selected at random one week prior to the experiment determined that 4 of the houses surveyed met the inclusion criteria; mean number of *Lu*. *longipalpis* = 35 (SE = 15, SD = 30). All experiments were undertaken within the ‘quintal’ (yard) area of the property where small bushes, some grass and fruit trees were characteristic of the vegetation. The chickens either roosted overnight in trees or a “shed”, constructed from wood, chicken wire, corrugated sheeting and any other construction materials available to the home owner. Some qualitative characteristics of the four study houses are summarised in Table 1.

**Table 1:** Additional characteristics of the houses.

House	Size of yard	Trees	Bushes	Grass	Dogs	Chickens (Quantity)	Other animals
**1**	Large	Y	N	Y	N	Y (10)	N
**2**	Medium	Y	Y	N	N	Y (15)	N
**3**	Medium	N	N	N	N	Y (15)	N
**4**	Large	Y	Y	N	Y	Y (15)	Y-parrots

Y = yes characteristic is present, N = no characteristic is not present. Large = >0.5 ha and medium = 0.1–0.5 ha.

The experiments took place between July and September 2016 which coincided with the end of winter and the beginning of summer, this period is characterised by dry and relatively cool weather (average rainfall <20mm and average nightly temperature 22°C). All experiments were conducted using modified Hoover Pugedo (HP) Centers for Disease Control (CDC) style light traps, without a light and suspended inside experimental chicken sheds [17]. Sand flies attracted to the HP trap were collected in nylon netting Barraud cages suspended beneath the trap. Experimental chicken sheds were made from 4 plywood panels (105cm long, 55cm wide), arranged in a square plan (55cm x 55cm). The panels were held together by cable-ties passed through holes (10 mm diameter) drilled in the top and bottom corners of each panel. A wooden dowel (20 mm diameter) placed across the top of the experimental chicken shed was used to suspend the HP trap inside the shed. A chicken, chosen at random from the householder’s chicken roost, was placed on the ground inside the experimental shed overnight. Pheromone lures, each containing 10mg of synthetic sex pheromone, were suspended from the underside of the lid of the HP trap [17].

Traps were set out at dusk between 15:00 and 17:00 hours local time. The netting cages and pheromone lures were removed from the HP traps the following morning between 07:00 and 09:00 hours and returned to the laboratory for examination. Sand flies were removed from the cage using a battery powered aspirator, placed in a -20°C freezer and numbers of both male and female *Lu*. *longipalpis* sand flies were determined by examination under a stereo-microscope (x20) (Quimis Q744S, SP, Brasil). Lures were placed in a freezer between sampling points to prevent loss of pheromone.

### Experiment 1: Attraction of Lu. longipalpis to different numbers of lures

The aim of the first experiment was to measure the effect of increasing the number of lures on *Lu*. *longipalpis* recruitment to pheromone treated sites. As such the experimental set-up used 1 pair of experimental chicken sheds per house. Each pair included a test shed and a control shed set 30 metres apart and each experimental shed contained a HP trap. Within a pair of sheds the position of the test and control were alternated each night to control for any potential positional bias. The pairs of traps were placed at 2 out of the 4 available household sites (e.g. the combination with 2-lures in the test shed vs. 1-lure in the control was performed in houses 1 and 2, the combination with 5-lures in the test shed vs. 1-lure in the control was carried out in houses 2 and 3, the combination with 10-lures in the test shed vs. 1-lure in the control was carried out in houses 3 and 4, the combination with 20-lures in the test shed vs. 1-lure in the control was carried out in houses 1 and 2, finally the combination with 50-lures in the test shed vs. 1-lure in the control was carried out in houses 3 and 4). The two houses used for each of these combinations of lures were chosen in a semi-recurring sampling design. All experimental sheds (control and test) were placed so that they were equidistant from the normal chicken roost (i.e. where the chickens normally roosted overnight). This resulted in the chickens’ normal roost being positioned mid-way between the test and control experimental chicken sheds. Chicken roosts were located ca. 5m from the house. Each combination was tested for 6 nights at each household location for a total of 60 trap nights (raw data provided in S1 Table).

### Experiment 2: Interaction between test and control traps placed 5, 10, 20 and 30m apart

The aim of the second experiment was to determine over what spatial scale competing experimental sheds interact. Using paired experimental chicken sheds, the test trap was baited with 5 lures and the control trap with 1 lure. Pairs of experimental chicken sheds were set out either 5, 10, 20 or 30 m apart and nightly numbers of male and female *Lu*. *longipalpis* captured determined as previously described. The combination with the test shed vs. control shed 5 m apart was performed in houses 1 and 3; 10 m apart was performed in houses 2 and 4; 20 m apart was performed in houses 1 and 3; 30m apart was performed in houses 2 and 3. Each distance between control and test traps (5, 10, 20, 30 m) was tested for 6 nights at each of two separate households (raw data provided in S2 Table) for a total of 48 trap nights. As in Experiment 1, the two houses used for each of these combinations of lures were chosen in a semi-recurring sampling design.

### Statistical analysis

In order to estimate 1) the attractiveness of different numbers of pheromone lures and 2) the interference between the test and control trap, we compared the number of sand flies caught by the test traps with those caught in the control traps for each respective experiment and employed a Bayesian log-linear model for analysis of contingency tables [21]. Bayesian analysis allows the calculation of posterior probabilities of the full model and its sub-models (allowing for covariate selection), independent of submodel size and structure, and therefore allows the inclusion of uncertainty into the inferential process [22].

Tables 2 and 3 in the Results section show the contingency tables prepared for the 2 experiments, summarising the sand fly catches for all possible combinations of house, trap type (test vs control) and experimental test condition (lure number or distance). As such, the total number of entries per contingency table (*y*_*i*_) is 40 for Experiment 1, and 32 for Experiment 2, including 0s for non-experimental nights (experimentally untested combinations). If we exclude experimentally untested combinations, data are reduced to 20 non-zero, usable entries for Experiment 1 and 16 for Experiment 2.

**Table 2:** Contingency table of total number of sand flies (and females only in parentheses) caught for each factor combination in Experiment 1, to test the number of pheromone lures per test trap.

	**Number of pheromone lures in the test traps**				
	***2***	***5***	***10***	***20***	***50***
**House**					
***1***	78 (19)	nd	nd	332 (74)	nd
***2***	117 (38)	243 (60)	nd	172 (66)	nd
***3***	nd	176 (47)	170 (34)	nd	300 (82)
***4***	nd	nd	528 (133)	nd	700 (153)
	**Number of pheromone lures in the control traps**				
	***1***	***1***	***1***	***1***	***1***
**House**					
***1***	58 (18)	nd	nd	31 (6)	nd
***2***	61 (25)	74 (18)	nd	47 (10)	nd
***3***	nd	58 (13)	43 (16)	nd	26 (5)
***4***	nd	nd	156 (40)	nd	170 (34)

The number of lures placed in the test traps was either 2, 5, 10, 20 or 50 lures. 1 lure was placed in the control trap. nd indicates that a collection was not done.

**Table 3:** Contingency table of total number of sand flies (and females only in parentheses) caught for each factor combination in Experiment 2 to test the interaction between pheromone baited traps over distance.

	Distance between test and control traps (m)			
	*5*	*10*	*20*	*30*
**test houses** (with 5 lures)				
***1***	154 (40)	nd	259 (62)	nd
***2***	nd	135 (33)	nd	243 (60)
***3***	71 (17)	nd	107 (18)	176 (47)
***4***	nd	313 (63)	nd	nd
**control houses** (with 1 lure)				
***1***	31 (11)	nd	60 (17)	nd
***2***	nd	31 (13)	nd	74 (18)
***3***	19 (4)	nd	48 (6)	58 (13)
***4***	nd	97 (18)	nd	nd

Distance between the test traps containing 5 lures and control traps containing 1 lure was 5, 10, 20 or 30m. nd indicates that a collection was not done.

In our Bayesian model, the contingency table entries, *y*_*i*_, are observations of independent Poisson random variables with mean *μ*_*i*_, *y*_*i*_ *~ P*(*μ*_*i*_), and likelihood:

$$l(\mu |y)=\prod_{i=1}^{n}\frac{1}{\mu_{i}!}\mu_{i}^{\mu_{i}}e^{-\mu_{i}}$$

with mean parameter *μ* modelled as

$$log(\mu_{i})=\beta x^{iT}=g^{i}$$

where *β* is the vector of (regression) coefficients for the dummy variables or indicators x^*i*^ of a given variable X (e.g. in the case of lures, the dummy variable for the 5-lure experimental condition will contain 1s for those entries associated with 5 lures and 0s for the others). Since *μ* has the same length of *y*, the model is saturated i.e. the number of parameters equals or is larger than the number of data entries (but see below for the solution).

The focus of this analysis was on the *β* coefficients, which are the measure of the association between the factors (household, trap type and test condition), their combinations (interaction), and the number of sand flies caught in traps. The meaning of the *β* coefficients in the log-linear model is identical to log odds ratios. The first step was, therefore, to assign a *prior* to the *β* coefficients that can allow identifiability of these parameters. Therefore, we assumed a noninformative Zellner’s *G*-prior (a multivariate normal distribution) [23] for the *β* coefficients of the indicator variables contained in **X**:

$${\beta \sim N_{i}(0_{i},\sigma^{2}(X}^{T}X{)}^{-1})$$

where *σ*^2^ is the scale parameter. The *β* posterior is:

$$p(\beta |y)\propto {\left|X^{T}X\right|}^{\frac{1}{2}}\Gamma (\frac{m}{2}){\left(\beta^{T}(X^{T}X)\beta \right)}^{-\frac{m}{2}}\pi^{-\frac{m}{2}}exp\{{\left(\sum_{i=1}^{n}y_{i}x^{i}\right)}^{T}\beta -\sum_{i=1}^{n}exp(x^{iT}\beta )\}$$

where *m* is the number of *β* parameters; *Γ* is the gamma function; and *π* is the Pi greco constant. The constant terms ${\left|X^{T}X\right|}^{\frac{1}{2}},\Gamma (\frac{m}{2})\;\text{and}\;\pi^{-\frac{m}{2}}$ were applied in the equation because they change according to the sub-model dimension (considered here in order to compare the importance of factor combinations in the full model).

The posterior distribution of *β* cannot be derived analytically. Samples from the posterior distribution of *β* were obtained by using a Markov Chain Monte Carlo (MCMC), an algorithm that allows the exploration of all the important regions of parameter-space. The MCMC algorithm is based on a random walk Metropolis-Hastings sampler proposed by Marin & Robert [23]. That is, initial *β* coefficients values and covariance matrix were obtained from a maximum likelihood estimation method. New *β* coefficients values are proposed from the *G*-prior using initial (later updated) *β* coefficients, a scale parameter (fixed at 0.5) and the fixed covariance matrix. Proposed *β* coefficients are accepted or rejected based on the log-likelihood ratio between *p*(*β*│**y**) with proposed *β* coefficients and *p*(*β*│**y**) with initial or updated *β* coefficients.

To apply the above model to the contingency Tables 2 and 3 (presented in the results section), we first converted the three factors (test/control, house, lures or distance) into indicators. We called the indicators *u* (test and control), *v* (house number) and *z* (number of lures in Experiment 1, or distance between experimental boxes in Experiment 2). *u* takes *L* values (i.e. 2), *v* takes *J* values (i.e. 4) and *z* takes *K* values (i.e. 5 in Experiment 1 or 4 in Experiment 2), so that the log-model for the mean parameter can be rewritten as:

$$log(\mu_{i(l,j,k)})=g+g_{l}^{u}+g_{j}^{v}+g_{k}^{z}+g_{lj}^{uv}+g_{lk}^{uz}+g_{jk}^{vz}+g_{ljk}^{uvz}$$

for *l* in 1,‥,*L*; *j* in 1,‥,*J*; and *k* in 1,‥,*K*.

where *g* is the reference average effect identifying marginal discrepancy for terms like $g_{l}^{u}$ or interaction discrepancy for terms like $g_{lj}^{uv}$.

In Experiment 1 (varying the number lures, with a constant distance between test and control), we assumed no three-factor interaction, i.e. $g_{ljk}^{uvz}=0$, and no interaction between house (*v*) and number of lures (*z*), i.e. $g_{jk}^{vz}=0$. The latter is equivalent to considering *v* and *z* conditionally independent given *u*. Therefore, the full model is:

$$log(\mu_{i(l,j,k)})=g+g_{l}^{u}+g_{j}^{v}+g_{k}^{z}+g_{lj}^{uv}+g_{lk}^{uz}$$

As seen in Tables 2 and 3, half of the comparisons are not made, therefore we have 20 entries for 30 parameters (saturated model, parameters coming from the dummy variables for test, house and number of lures, and permitted interactions between house x test, house x number lures, test x number of lures) in experiment 1. We have 16 entries for 27 parameters in experiment 2 (dummy variables for test, house and distance, and permitted interactions between house x test, house x distances, test x distances). To ensure identifiability of the parameters, which makes the ANOVA comparison between the full model and its sub-model feasible, constraints are imposed. By setting to zero the parameters corresponding to the first category of each variable (excluded category):

$$g_{1}^{u}=g_{1}^{v}=g_{1}^{z}=g_{11}^{uv}=g_{12}^{uv}=g_{21}^{uv}=g_{13}^{uv}=g_{14}^{uv}=g_{11}^{uz}=g_{12}^{uz}=g_{21}^{uz}=g_{13}^{uz}=g_{14}^{uz}=g_{15}^{uz}=0$$

the saturated model becomes non-saturated (20 entries for 16 parameters).

In Experiment 2 (varying distance between test and control traps with a constant number of lures), we again assumed no three-factor interaction, $g_{ljk}^{uvz}=0$, and no interaction between house (*v*) and distance between chicken shed (*z*), $g_{jk}^{vz}=0$. Again, since half of the comparisons are not made the number of entries is 16 for 27 parameters. By setting to zero the parameters corresponding to the first category of each variable:

$$g_{1}^{u}=g_{1}^{v}=g_{1}^{z}=g_{11}^{uv}=g_{12}^{uv}=g_{21}^{uv}=g_{13}^{uv}=g_{14}^{uv}=g_{11}^{uz}=g_{12}^{uz}=g_{21}^{uz}=g_{13}^{uz}=g_{14}^{uz}=0$$

the total number of parameters is reduced to 14 for 16 entries (non-saturated model).

The sub-models considered for Experiment 1 are:

$log(\mu_{i(l,j,k)})=g+g_{l}^{u}+g_{j}^{v}+g_{k}^{z}+g_{lk}^{uz}$ without interaction test/control and house number

$log(\mu_{i(l,j,k)})=g+g_{l}^{u}+g_{j}^{v}+g_{k}^{z}+g_{lj}^{uv}$ without interaction test/control and number of lures

and for Experiment 2:

$log(\mu_{i(l,j,k)})=g+g_{l}^{u}+g_{j}^{v}+g_{k}^{z}+g_{lk}^{uz}$ without interaction test/control and house number

$log(\mu_{i(l,j,k)})=g+g_{l}^{u}+g_{j}^{v}+g_{k}^{z}+g_{lj}^{uv}$ without interaction test/control and distance between chicken sheds.

Comparisons between full model and submodels (full model in which an interaction between factors is removed) are made by calculating the Bayes factor [24]:

$$BF=\frac{likelihood\;full\;model}{likelihood\;submodel}$$

We then take the log_10_(1/*BF*) [25] and interpret this value by using Jeffrey’s scale of evidence [26]: if log(1/*BF*) is larger than 1, we consider the removed interaction to be significant in the full model (the extended scale of evidence is: removed interaction anecdotal for values <0.5; substantial for values between 0.5–1; strong for values between 1 and 2; and decisive for values > 2).

The statistical analysis was repeated for the data in both Tables 2 and 3 and calculated for the number of caught male sand flies, number of caught female sand flies and total (male+female) sand flies. The analysis was performed in R-cran software [27], specifically using the Bayes package [28] for the MCMC algorithm calculations (functions *hmnoinfloglin* and *loglinnoinflpost*).

### Ethics statement

The project, including the involvement of householders, was reviewed and approved by the Faculty of Health and Medicine Ethical Review Committee (FHMREC15125) at Lancaster University. Consent was obtained from the Governador Valadares district health authority Centro de Controle de Zoonoses (CCZ) to conduct the study within their administrative jurisdiction. This study was carried out in accordance with the guidelines of the Animals in Science Regulation Unit (ASRU) and in compliance with the Animals (Scientific Procedures) Act (ASPA) 1986 (amended 2012) regulations and was consistent with UK Animal Welfare Act 2006 and The Welfare of Farmed Animals (England) Regulations 2007 and 2010.

## Results

The results of the sand fly captures are presented in contingency tables (Tables 2 and 3) for each experiment. The raw data is presented in S1 and S2 Tables respectively.

### Model fitting

The models for experiment 1 and 2 were run for 100,000 iterations to evaluate if the MCMC engine converged (in other words, if we reached a stable configuration of the posterior and its parameters). Thus, we investigated the MCMC traces and histograms generated in the Bayesian analysis. The MCMC traces for each *β* coefficient, and the histograms for each *β* posterior distribution for experiment 1 and 2 are provided in supplementary files S1, S2, S3, S4 and S5 Figs. Convergence statistics for experiment 1 are shown in Table 4 (and in supplementary file S3 Table for experiment 2, where distance 5m and house 1 were the reference variables), where for the last 30,000 iterations divided in 3 blocks of 10,000 iterations, the variations in the posterior mean and posterior variance are presented. These values are small and guarantee stable posterior histograms for each coefficient. The overall conclusion is that the models for experiment 1 and experiment 2 converged to stable posteriors for all the parameters. This guarantees a good approximation of the credible intervals (CR) for all the parameters.

**Table 4:** Experiment 1. *β* coefficients convergence.

Coeff.	Mean1	Mean2	Mean3	Diff(%)	Var1	Var2	Var3	Diff(%)
**ßInter**	3.968	3.988	3.979	0.488	0.014	0.014	0.014	3.477
**ßtest**	0.771	0.751	0.757	2.708	0.019	0.019	0.019	3.376
**ßh2**	0.204	0.187	0.190	9.216	0.020	0.021	0.022	9.781
**ßh3**	-0.057	-0.065	-0.072	22.372	0.056	0.052	0.058	10.602
**ßh4**	1.514	1.487	1.486	1.858	0.073	0.064	0.063	14.717
**ßl5**	0.138	0.117	0.135	15.811	0.028	0.027	0.027	5.479
**ßl10**	-0.382	-0.377	-0.363	5.227	0.063	0.054	0.053	17.001
**ßl20**	-0.406	-0.434	-0.411	6.562	0.018	0.020	0.018	10.775
**ßl50**	-0.398	-0.392	-0.394	1.501	0.060	0.058	0.054	10.053
**ßch2**	-0.539	-0.534	-0.538	0.986	0.027	0.026	0.027	1.852
**ßch3**	-0.610	-0.602	-0.616	2.177	0.072	0.071	0.069	3.567
**ßch4**	-1.223	-1.185	-1.210	3.194	0.091	0.085	0.075	19.641
**ßcl5**	0.951	0.978	0.973	2.810	0.040	0.039	0.039	4.221
**ßcl10**	1.577	1.558	1.573	1.245	0.081	0.077	0.068	16.898
**ßcl20**	1.348	1.380	1.364	2.357	0.024	0.027	0.025	10.372
**ßcl50**	1.952	1.937	1.966	1.479	0.078	0.081	0.064	22.983

For each coefficient the mean and variance are reported for the last three MCMC sub-chains (e.g. from 70,001 to 80,000; from 80,001 to 90,000; and from 90,001 to 100,000). Diff (%) is the proportion of variation in the mean and variance compared to the mean of the means and the mean of the variances. Inter, is the intercept; test is the variable containing test and controls (0 for controls and 1 for tests); ch is the interaction between test and house; cl is the interaction between test and pheromones; h is the house (house number 2, 3 and 4, reference house number 1); and l is the number of pheromone lures (5, 10, 20, 50, reference 2 lures).

### Experiment 1: Attraction of Lu. longipalpis to different numbers of lures

Mean, variance and CR of the *β* coefficients for the last 30,000 iterations are shown in Table 5. This table shows that the interaction between test and control traps and number of pheromone lures are all significantly different from zero (ßcl5, ßcl10, ßcl20, ßcl50) despite some non-interactive term not being significantly different from zero (shaded rows) (house 2 and 3 and lure numbers 5 and 10). Overall, differences in catches between test and control traps were positively associated with the different numbers of pheromone lures deployed in the trap.

**Table 5:** *β* coefficients summary statistics from the posterior distributions (last 10,000 iterations).

Coeff.	Mean	Variance	CR.0.05	CR.0.95
**ßInter**	3.968	0.014	3.775	4.160
**ßtest**	0.771	0.019	0.533	1.005
**ßh2**	0.204	0.020	-0.038	0.429
**ßh3**	-0.057	0.056	-0.454	0.343
**ßh4**	1.514	0.073	1.070	1.939
**ßl5**	0.138	0.028	-0.133	0.422
**ßl10**	-0.382	0.063	-0.790	0.034
**ßl20**	-0.406	0.018	-0.634	-0.186
**ßl50**	-0.398	0.060	-0.785	-0.001
**ßch2**	-0.539	0.027	-0.803	-0.273
**ßch3**	-0.610	0.072	-1.057	-0.188
**ßch4**	-1.223	0.091	-1.721	-0.742
**ßcl5**	0.951	0.040	0.614	1.282
**ßcl10**	1.577	0.081	1.131	2.036
**ßcl20**	1.348	0.024	1.089	1.614
**ßcl50**	1.952	0.078	1.500	2.410

Reference factors: 2 lures and house 1

CR is the credible interval. Inter, is the intercept; test is the variable containing test and controls (0 for controls and 1 for tests); ch is the interaction between test and house; cl is the interaction between test and pheromones; h is the house (house number 2, 3 and 4); and l is the number of pheromone lures. Shaded areas indicate non-significant interactions (i.e. the credible interval crosses 0).

In the ANOVA comparison between the full model and its components, by adopting the Jeffrey’s scale of evidence (see Methods) the interaction between house number and test and control is substantial (log_10_(1/*BF*) = 0.73), while the interaction between number of lures and test and control is decisive (log_10_(1/*BF*) = 14.65).

In addition, and to test our initial assumption that there was conditional independence between house number and level of pheromone (i.e. house characteristics are not influenced by pheromones and vice versa) we ran sub-models containing only a single interaction, and we found that only the interaction between test and control and number of lures is decisive (log_10_(1/*BF*) = 12.01), compared to test and control and house number (log_10_(1/*BF*) = 0.05) and house number and number of lures (log_10_(1/*BF*) = 0.0001) (which for Jeffrey’s scale of evidence are considered anecdotal).

Given the results shown in Tables 4 and 5, we were able to restrict the full model to contain only the interaction between test and control and number of lures (largest coefficients and significantly different from 0 for all the houses). The restricted (parsimonious) model, includes coefficients for the indicators for the number of lures and indicators of the interactions between test and control and number of lures are reported in Table 6 (and histograms in supplementary information S5 Fig).

**Table 6:** *β* coefficients summary statistics from the posterior distributions (last 10,000 iterations) of number of pheromone lures, and interaction between test and controls and level of pheromones.

Coeff.	Mean	Variance	CR.0.05	CR.0.95
**ßl2**	4.080	0.008	3.927	4.223
**ßl5**	4.185	0.007	4.035	4.324
**ßl10**	4.597	0.005	4.477	4.709
**ßl20**	3.655	0.013	3.462	3.837
**ßl50**	4.583	0.005	4.465	4.697
**ßcl2**	0.494	0.013	0.308	0.690
**ßcl5**	1.157	0.009	1.000	1.325
**ßcl10**	1.258	0.006	1.125	1.392
**ßcl20**	1.871	0.014	1.675	2.072
**ßcl50**	1.631	0.005	1.506	1.765

**Fig 2:**
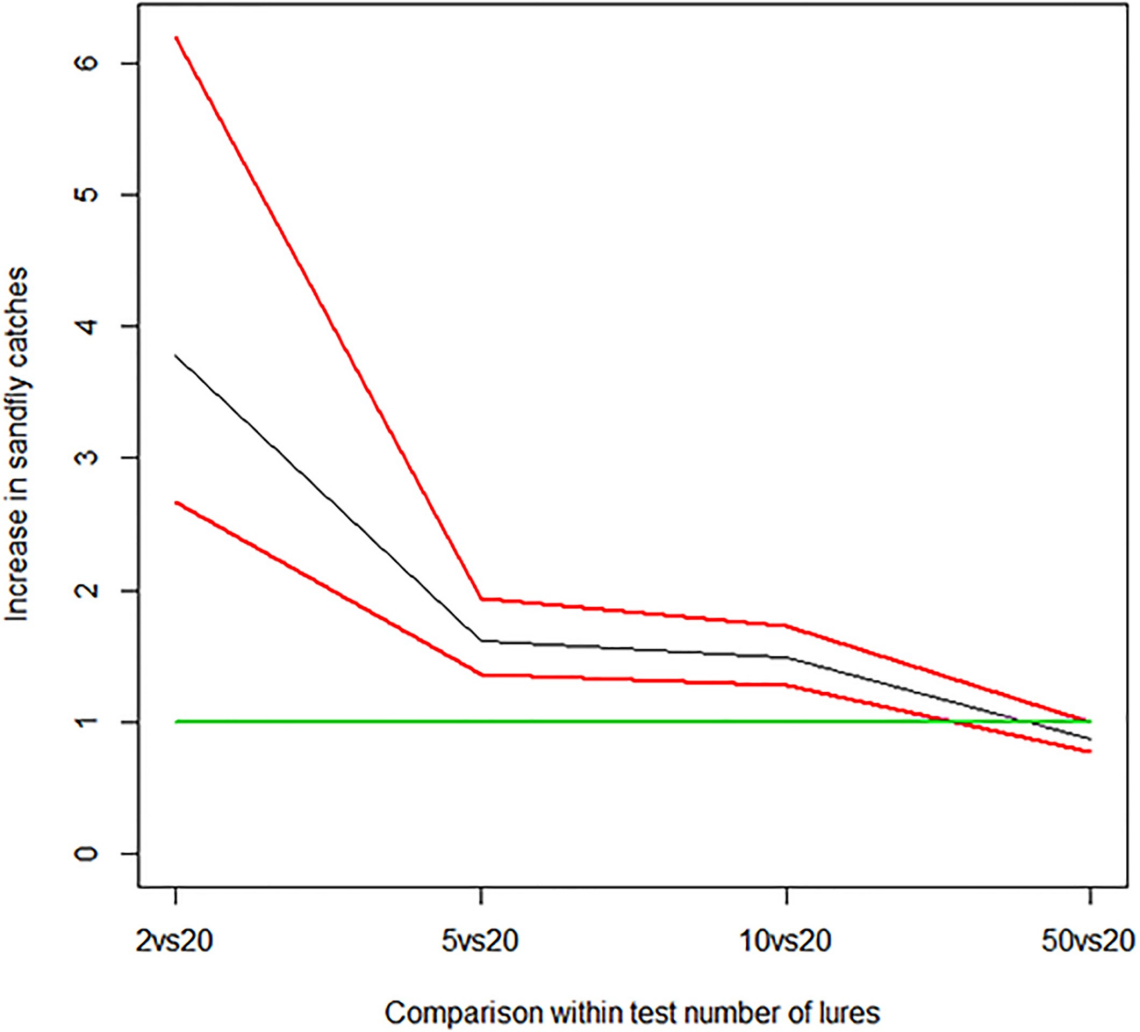
Comparison of the *β* coefficients for the different levels of pheromones. The 95% CR limits are shown in red. The green line is the ratio of 1 (identical coefficients, no change in capture rate).

Table 6 shows that all the coefficients for the pheromone model are significantly different from 0. Table 7 shows that in all the comparison between lures, 20 lures has the largest ratio. A comparison between the coefficients of the interactions (Table 6 and Fig 2) shows the greatest increase in capture rate (relatively larger coefficient) from 2 lures (2.6–6.2, 95% CR, 2vs20 in Fig 2) to 20 lures (indicated with *β*cl20 in Table 6), which represents a 3.8-fold increment in total sand fly capture as lures are increased 10-fold. By comparison, the change in coefficient from 20 and 50 lures indicates the capture rates are not significantly increased.

**Table 7:** Ratio of the *β* coefficients for the levels of pheromones and 95% credible intervals.

Lures comparison		Lower CR	Median Ratio	Upper CR
5	2	1.61	2.33	3.88
10	2	1.79	2.53	4.19
**20**	**2**	**2.66**	**3.78**	**6.19**
50	2	2.33	3.29	5.42
10	5	0.91	1.08	1.31
**20**	**5**	**1.36**	**1.61**	**1.93**
50	5	1.21	1.41	1.67
**20**	**10**	**1.28**	**1.48**	**1.72**
50	10	1.13	1.29	1.48
50	20	0.76	0.87	0.99

In the cross-comparison between numbers of lures (Table 7), statistically significant increases (when both the CR limits are above one) were found for all the comparisons apart from between 10 and 5 lures or between 20 and 50 lures.

### Experiment 1: Attraction of male and female Lu. longipalpis to different numbers of lures

The interaction between test and control and house number has a log_10_(1/*BF*) = 0.92 (substantial for the Jeffrey’s scale of evidence) for male and 0.001 (anecdotal) for female sand flies; while the interaction between test and control and number of lures has a log_10_(1/*BF*) = 6.40 (decisive) for male and 5.56 (decisive) for female sand flies. This means that even when considering the sand flies by sex, the house has little effect on the number of catches, while the pheromone quantity influences both male and females sand fly trap catches, with a slight preference for male sand flies (however this may also reflect the proportion of male/female in the sand fly populations). In fact, even if female sand flies show the largest fold increase from 2 lures to 20 lures (5.9), this is statistically insignificant (-33.1–41.2, 95%CR). Table with numbers of male and female sand flies is shown in S4 Table.

### Experiment 2: Total, male and female sand fly counts in test and control traps placed 5, 10, 20 and 30m apart

In this experiment, the numbers of male, female and total sand flies attracted to test (5 lures) and control (1 lure) experimental sheds, were investigated at different distances (5, 10, 20 and 30m) between sheds. No significant relationship was found for either male, female or total sand flies when modelled with test and control, house and distance variables (taking as reference variables the distance at 5m and house number 1). In particular:

Anecdotal interaction between test and control and house number, log_10_(1/*BF*) = 0.004 for total number of sand flies; 0.0009 for male and 0.103 for female sand flies;

Anecdotal interaction between test and control and distance between experimental boxes, log_10_(1/*BF*) = 0.0002 for total number of sand flies; 0.001 for male and 0.001 for female sand flies.

Experiment 2 results show that the distance between the test and control chicken sheds does not influence the number of sand flies caught in the test or control traps. Instead the numbers of sand flies caught is determined by the number of lures used in the trap, as the difference between test and control trap catches was significant at all the distances apart (confirming results above). ß coefficient for the dummy variable test representing 5 lures is equal to 1.7 (1.39, 2.02, CR) for the total number of sand flies, 1.3 (0.76, 2.05, CR) for female sand flies, and finally 1.8 (1.43, 2.22, CR) for male sand flies. These coefficients do not change significantly when considering the same model applied to each distance individually: median difference from 1.7 of -0,03 (-0.21,0.16 CR) for the total number of sand flies, median difference from 1.3 of 0.03 (-0.34, 0.41, CR) for female sand flies, and finally median difference from 1.8 of -0.04 (-0.26, 0.16, CR). The results suggest that differences between test and control are similar at the different distances and that increasing the separation of the test and control traps does not favour trapping either males or females. This finding seems to indicate that the trapping is operating within a spatially homogeneous sand fly population in the peridomestic environment. If the sand fly population was spatially heterogeneous we might expect the proportion of flies caught in the 1-lure traps to 5-lure traps to change substantially as the distance between the traps changed.

## Discussion

This is the first time that a Bayesian log-linear model has been employed to quantify exogenous effects on sand fly catches. The model used allowed analysis of the contingency table obtained from multiple concurrent experiments containing categorical variables only, to identify the most important factors affecting the number of sand fly catches and to include model and data uncertainty in the model inference. Classically, analyses using simple (deterministic) log-linear models of sand fly count data are applied (e.g. [29, 30]), this can lead to limited interpretation of the *β* coefficients, and therefore of the effect of each factor, since a measure of uncertainty is missing. In addition, simple (deterministic) log-linear models do not allow for a comparison between the distributions (the values) of the *β* coefficients, which allows us to obtain credible interval data from differences between two *β* coefficients (Fig 2).

To our knowledge, a similar approach has only been applied once before in a study that examined the differences between human landing catches and light trap catches for capture of *Anopheles gambiae* [31]. However, that publication did not account for the interaction between levels within and between each variable, leaving the method (and analysis) not fully exploited. Our approach considered the interactions between factors and therefore allowed us to dissect the effect of each factor on the full model and consequently view the outcomes with a high degree of certainty.

The first experiment showed that increasing the number of lures increased the total number of *Lu*. *longipalpis* (both males and females) caught by the traps (Table 2). Overall, we collected more male than female sand flies confirming previous observations, using a variety of different trap types in GV and elsewhere, that suggest that there may be more male than female *Lu*. *longipalpis* in the population [6, 15–17]. A group of 20 or 50 pheromone lures was significantly more attractive than groups of 2, 5 or 10 lures (Table 6).

Overall the increase in catch was not proportional to the increase in the number of lures i.e. the number of *Lu*. *longipalpis* caught increased in steps (Fig 2) as the number of lures increased. Increasing the quantity of pheromone lures is equivalent to increasing the release rate of the sex pheromone and therefore the effective distance at which the insects can detect the pheromone, it follows therefore, that more sand flies should be caught with higher quantities of pheromone than with lower quantities, as the pheromone would be able to attract sand flies from further away [32]. However, in this study, the relative contribution of additional lures to total catch reached a plateau: 50 lures were not significantly more attractive than 20 lures (after the effect of houses on trap catches is accounted for). It is also possible that at very high release rates the pheromone is repellent [33].

When the proximity between the test and control traps, (but not the quantity of pheromone), varied, we found that the proximity of the test traps (5 lures) had no effect on the control trap catches (1 lure) even when the test and control traps were only 5 m apart. At all the distances tested, the traps with 5 lures were significantly more attractive than the traps with 1 lure (confirming previous result of experiment 1), but critically, that this difference in capture rate (in both absolute and relative terms) between 5 lures and 1 lure was consistent over the tested distances. Thus, at distances equal to or greater than 5 m neighbouring traps do not appear to affect each other’s attractiveness, at least when the number of lures used is relatively small. Indicating no contamination between test and control traps over distances used in the established protocol.

The differences between the test and control traps were consistent and significant at all the distances tested, therefore it is unlikely that the two traps are combining their effect. Although the role of the male pheromone as an attractant and in forming aggregations is well established [14,15,16,34], this result may indicate the important role of the pheromone in maintaining aggregations. The results show that the sand flies are attracted to the area of the pheromone release in proportion to the amount of pheromone present and once in the vicinity of the pheromone the HP trap samples that population. The results suggest that the sand flies are aggregated (or arrested) at that site because the proportion of flies collected in the 1-lure traps compared to the 5-lure traps is similar regardless of how far apart they are placed. However, in these experiments once the sand flies enter the trap they cannot migrate towards the area of greater pheromone concentration. Therefore, we cannot be sure that eventually all sand flies would not move towards the site of greater pheromone release. In the future we could test this hypothesis using a mark release recapture experiment. It would be interesting to determine the minimum amount of pheromone required to maintain aggregations and if interaction between pheromone sources occurs at distances less than 5m. There is also a possibility that males captured in the Barraud cages would attract other males and females thus adding a source of error. We discount this as a major source of error for either experiment because of the small numbers of males involved compared to the pheromone released by the lures. An implication of this result is that naturally established aggregations would compete with synthetic pheromone lure-and-kill sites. However, little is known about the stability, longevity or density of naturally established aggregation sites [14] however the lure-and-kill sites would be active for long periods of time and present in greater numbers than untreated sites where aggregations are known to form. It could also be the case that the attractiveness of sites treated with synthetic pheromone lures would be enhanced by the presence of real male *L*. *longipalpis* sex aggregation pheromone.

The results also suggest the possible existence of a spatially homogenous *Lu*. *longipalpis* population in the peridomestic environment. This is an interesting possibility requiring further work to clarify the situation. Previous work using CDC miniature light traps has suggested that *Lu*. *longipalpis* is heterogeneously distributed and that adult males and females aggregate sporadically in chicken sheds and other animal shelters. However, it is not understood why, in an area with many potential aggregation sites, aggregations appear to develop at some sites but others. Our historical understanding of *Lu*. *longipalpis* distribution may be related to the use of miniature CDC light traps which possibly may not be adequate for sampling the *Lu*. *longipalpis* population.

Taken together the results of experiment 1 and 2 suggest that the most effective way to use the pheromone would be to use as many lures as possible distributed widely in any given area rather than use the same number of lures grouped together in a small number of places within the same area. Increasing the number of lures and thereby increasing the release rate of pheromone would also increase trap catches but clearly there is a trade-off between the number of lures used, their cost and effectiveness.

The effect of the house location was substantial but not decisive (apart from house 4, the only one with a significant coefficient when considering the full model), in other words, house conditions slightly improve the model and therefore additional studies should focus on which factors, related to the house, influence the overall catches in order to be taken into account in future analyses. It has been shown that sand flies, in particular *Lu*. *longipalpis*, favour humid environments with lots of vegetation [35]. Habitat-specific effects on pheromone attraction is also a potentially important factor playing a critical role in shaping the response to pheromones [36].

This study showed that increasing the number of pheromone lures increased the numbers of sand flies captured. This is not a linear relationship and increasing the number of lures by a given factor does not lead to a similar increase in the number of sand flies caught. However, in the context of a control programme, greater numbers of pheromone lures placed next to an insecticide sprayed wall, would result in more sand flies being killed and potentially a more effective control programme. The second experiment highlighted the attraction and possible aggregation behaviour by *Lu*. *longipalpis* once a pheromone source had been located. In the context of a control program this result suggests that the sand flies would remain strongly aggregated at the synthetic pheromone insecticide treated site and that “lure-and-kill” sites even when positioned in relatively close proximity would not compete with each other.

## Supporting information

S1 Table Raw data from Experiment 1—experimental traps treated with 2, 5, 10, 20 or 50 lures, control traps = 1 lure. Distance between test and control traps = 30m.
(PDF)

S2 Table Raw data from Experiment 2 traps set 5, 10, 20 or 30 m apart, control trap = 1 lure, test trap = 5 lures. (PDF)

S3 Table Experiment 2. *β* coefficients convergence. For each coefficient the mean and variance are reported for the last three MCMC sub-chains (e.g. from 70,001 to 80,000; from 80,001 to 90,000; and from 90,001 to 100,000). Diff (%) is the proportion of variation in the mean and variance compared to the mean of the means and the mean of the variances. Inter, is the intercept; test is the variable containing test and controls (0 for controls and 1 for tests); ch is the interaction between test and house; cd is the interaction between test and distance; h is the house (house number 2, 3 and 4); and d is the distance (10m, 20m and 30m).
(PDF)

S4 Table Experiment 2. Male (A) and Female (B) response to different numbers of lures.
(PDF)

S1 Fig Experiment 1. MCMC traces for the *β* coefficients. Explanation of titles after the *β*: **inter**, is the intercept of the model; **test** is the variable containing test and controls (0 for controls and 1 for tests); **ch** is the interaction between test and house; **cl** is the interaction between test and pheromones; **h** is house (house number 2, 3 and 4); and **l** refers to pheromone lures (2, for 5 lures; 3 for 10 lures; 4 for 20 lures; and 5 for 50 lures).
(PDF)

S2 Fig Experiment 2. MCMC traces for the *β* coefficients. Explanation of titles after the *β*: **inter**, is the intercept of the model; **test** is the variable containing test and controls (0 for controls and 1 for tests); **ch** is the interaction between test and house; **cd** is the interaction between test and distance; **h** is house (house number 2, 3 and 4); and **d** refers to distances (10m, 20m, 30m).
(PDF)

S3 Fig Experiment 1. *β* coefficients histograms from the posterior distributions. *β*: **inter** is the model intercept; **test** is the variable containing test and controls (0 for controls and 1 for tests); **ch** is the interaction between test and house; **cl** is the interaction between test and pheromones; **h** is referred to house (house number 2, 3 and 4); and **l** is referred to pheromone lures (2, for 5 lures; 3 for 10 lures; 4 for 20 lures; and 5 for 50 lures).
(PDF)

S4 Fig Experiment 2. *β* coefficients histograms from the posterior distributions. Explanation of titles after the *β*: **inter**, is the intercept of the model; **test** is the variable containing test and controls (0 for controls and 1 for tests); **ch** is the interaction between test and house; **cd** is the interaction between test and distance; **h** is house (house number 2, 3 and 4); and **d** refers to distances (10m, 20m, 30m).
(PDF)

S5 Fig Experiment 1. *β* coefficients histograms from the posterior distributions of the number of pheromone lures and their interaction with test/control. Explanation of titles: l is the number of pheromone lures (1 is 2 lures; 2 is 5 lures; 3 is 10 lures; 4 is 20 ures; and 5 is 50 lures); cl is the interaction between test and pheromone.
(PDF)
