# Supplementary material for: Attraction of Lutzomyia longipalpis to synthetic sex-aggregation pheromone: Effect of release rate and proximity of adjacent pheromone sources
Source: PLoS Negl Trop Dis. 2018 Dec 19;12(12):e0007007. doi: 10.1371/journal.pntd.0007007 (PMC6300254; doi:10.1371/journal.pntd.0007007)
Supplement: S1 Table — Distance between test and control traps = 30m. (PDF) [file pntd.0007007.s001.pdf]

S1 Table

Raw data from Experiment 1 - experimental traps treated with 2, 5, 10, 20 or 50 lures, cont

Distance between test and control traps = 30m.

Date	replicate	HHID	Name	Position	distance apart	Treatment	Males
08/08/2016	1	1	Leonidia	A	30m	1 pheromone	6
08/08/2016	1	1	Leonidia	B	30m	2 pheromone	14
08/08/2016	1	2	Arlan	A	30m	1 pheromone	4
08/08/2016	1	2	Arlan	B	30m	2 pheromone	29
09/08/2016	2	1	Leonidia	A	30m	2 pheromone	11
09/08/2016	2	1	Leonidia	B	30m	1 pheromone	0
09/08/2016	2	2	Arlan	A	30m	2 pheromone	3
09/08/2016	2	2	Arlan	B	30m	1 pheromone	9
10/08/2016	3	1	Leonidia	A	30m	1 pheromone	9
10/08/2016	3	1	Leonidia	B	30m	2 pheromone	15
10/08/2016	3	2	Arlan	A	30m	1 pheromone	3
10/08/2016	3	2	Arlan	B	30m	2 pheromone	19
11/08/2016	4	1	Leonidia	A	30m	2 pheromone	5
11/08/2016	4	1	Leonidia	B	30m	1 pheromone	2
11/08/2016	4	2	Arlan	A	30m	2 pheromone	6
11/08/2016	4	2	Arlan	B	30m	1 pheromone	10
15/08/2016	5	1	Leonidia	A	30m	1 pheromone	0
15/08/2016	5	1	Leonidia	B	30m	2 pheromone	4
15/08/2016	5	2	Arlan	A	30m	1 pheromone	5
15/08/2016	5	2	Arlan	B	30m	2 pheromone	11
16/08/2016	6	1	Leonidia	A	30m	2 pheromone	10
16/08/2016	6	1	Leonidia	B	30m	1 pheromone	23
16/08/2016	6	2	Arlan	A	30m	2 pheromone	11
16/08/2016	6	2	Arlan	B	30m	1 pheromone	5

05/09/2016	1	3	Marcelo	A	30m	1 pheromone	3
05/09/2016	1	3	Marcelo	B	30m	5 pheromone	42
05/09/2016	1	2	Arlan	A	30m	1 pheromone	9
05/09/2016	1	2	Arlan	B	30m	5 pheromone	34
06/09/2016	2	3	Marcelo	A	30m	5 pheromone	3
06/09/2016	2	3	Marcelo	B	30m	1 pheromone	14
06/09/2016	2	2	Arlan	A	30m	5 pheromone	23
06/09/2016	2	2	Arlan	B	30m	1 pheromone	7
07/09/2016	3	3	Marcelo	A	30m	1 pheromone	3
07/09/2016	3	3	Marcelo	B	30m	5 pheromone	48
07/09/2016	3	2	Arlan	A	30m	1 pheromone	13
07/09/2016	3	2	Arlan	B	30m	5 pheromone	41
08/09/2016	4	3	Marcelo	A	30m	5 pheromone	1
08/09/2016	4	3	Marcelo	B	30m	1 pheromone	22
08/09/2016	4	2	Arlan	A	30m	1 pheromone	12
08/09/2016	4	2	Arlan	B	30m	5 pheromone	36
12/09/2016	5	3	Marcelo	A	30m	1 pheromone	1
12/09/2016	5	3	Marcelo	B	30m	5 pheromone	29

12/09/2016	5	2	Arlan	A	30m	5 pheromone	22
12/09/2016	5	2	Arlan	B	30m	1 pheromone	8
13/09/2016	6	3	Marcelo	A	30m	5 pheromone	6
13/09/2016	6	3	Marcelo	B	30m	1 pheromone	2
13/09/2016	6	2	Arlan	A	30m	5 pheromone	27
13/09/2016	6	2	Arlan	B	30m	1 pheromone	7

27/07/2016	1	3	Marcelo	A	30m	10 pheromone	8
27/07/2016	1	3	Marcelo	B	30m	1 pheromone	6
27/07/2016	1	4	Carlos	A	30m	10 pheromone	171
27/07/2016	1	4	Carlos	B	30m	1 pheromone	19
28/07/2016	2	3	Marcelo	A	30m	1 pheromone	5
28/07/2016	2	3	Marcelo	B	30m	10 pheromone	60
28/07/2016	2	4	Carlos	A	30m	1 pheromone	38
28/07/2016	2	4	Carlos	B	30m	10 pheromone	93
01/08/2016	3	3	Marcelo	A	30m	10 pheromone	6
01/08/2016	3	3	Marcelo	B	30m	1 pheromone	2
01/08/2016	3	4	Carlos	A	30m	10 pheromone	45
01/08/2016	3	4	Carlos	B	30m	1 pheromone	27
02/08/2016	4	3	Marcelo	A	30m	1 pheromone	0
02/08/2016	4	3	Marcelo	B	30m	10 pheromone	25
02/08/2016	4	4	Carlos	A	30m	1 pheromone	16
02/08/2016	4	4	Carlos	B	30m	10 pheromone	4
03/08/2016	5	3	Marcelo	A	30m	10 pheromone	1
03/08/2016	5	3	Marcelo	B	30m	1 pheromone	14
03/08/2016	5	4	Carlos	A	30m	10 pheromone	65
03/08/2016	5	4	Carlos	B	30m	1 pheromone	4
04/08/2016	6	3	Marcelo	A	30m	1 pheromone	0
04/08/2016	6	3	Marcelo	B	30m	10 pheromone	36
04/08/2016	6	4	Carlos	A	30m	1 pheromone	12
04/08/2016	6	4	Carlos	B	30m	10 pheromone	17

27/07/2016	1	1	Leonidia	A	30m	20 pheromone	25
27/07/2016	1	1	Leonidia	B	30m	1 pheromone	6
27/07/2016	1	2	Arlan	A	30m	20 pheromone	12
27/07/2016	1	2	Arlan	B	30m	1 pheromone	11
28/07/2016	2	1	Leonidia	A	30m	1 pheromone	3
28/07/2016	2	1	Leonidia	B	30m	20 pheromone	47
28/07/2016	2	2	Arlan	A	30m	1 pheromone	2
28/07/2016	2	2	Arlan	B	30m	20 pheromone	16
01/08/2016	3	1	Leonidia	A	30m	20 pheromone	17
01/08/2016	3	1	Leonidia	B	30m	1 pheromone	4
01/08/2016	3	2	Arlan	A	30m	20 pheromone	10
01/08/2016	3	2	Arlan	B	30m	1 pheromone	14
02/08/2016	4	1	Leonidia	A	30m	1 pheromone	6
02/08/2016	4	1	Leonidia	B	30m	20 pheromone	74
03/08/2016	4	2	Arlan	A	30m	20 pheromone	6
03/08/2016	4	2	Arlan	B	30m	1 pheromone	6
03/08/2016	5	1	Leonidia	A	30m	20 pheromone	58
03/08/2016	5	1	Leonidia	B	30m	1 pheromone	3
04/08/2016	5	2	Arlan	A	30m	1 pheromone	2
04/08/2016	5	2	Arlan	B	30m	20 pheromone	24

04/08/2016	6	1	Leonidia	A	30m	1 pheromone	3
04/08/2016	6	1	Leonidia	B	30m	20 pheromone	37
05/08/2016	6	2	Arlan	A	30m	20 pheromone	38
05/08/2016	6	2	Arlan	B	30m	1 pheromone	2

08/08/2016	1	3	Marcelo	A	30m	1 pheromone	1
08/08/2016	1	3	Marcelo	B	30m	50 pheromone	83
08/08/2016	1	4	Carlos	A	30m	1 pheromone	56
08/08/2016	1	4	Carlos	B	30m	50 pheromone	85
09/08/2016	2	3	Marcelo	A	30m	50 pheromone	2
09/08/2016	2	3	Marcelo	B	30m	1 pheromone	8
09/08/2016	2	4	Carlos	A	30m	50 pheromone	88
09/08/2016	2	4	Carlos	B	30m	1 pheromone	1
10/08/2016	3	3	Marcelo	A	30m	1 pheromone	2
10/08/2016	3	3	Marcelo	B	30m	50 pheromone	51
10/08/2016	3	4	Carlos	A	30m	1 pheromone	41
10/08/2016	3	4	Carlos	B	30m	50 pheromone	73
11/08/2016	4	3	Marcelo	A	30m	50 pheromone	4
11/08/2016	4	3	Marcelo	B	30m	1 pheromone	8
11/08/2016	4	4	Carlos	A	30m	50 pheromone	76
11/08/2016	4	4	Carlos	B	30m	1 pheromone	4
15/08/2016	5	3	Marcelo	A	30m	1 pheromone	0
15/08/2016	5	3	Marcelo	B	30m	50 pheromone	54
15/08/2016	5	4	Carlos	A	30m	1 pheromone	30
15/08/2016	5	4	Carlos	B	30m	50 pheromone	109
16/08/2016	6	3	Marcelo	A	30m	50 pheromone	24
16/08/2016	6	3	Marcelo	B	30m	1 pheromone	2
16/08/2016	6	4	Carlos	A	30m	50 pheromone	116
16/08/2016	6	4	Carlos	B	30m	1 pheromone	4

rol traps = 1 lure.

Females	Total
5	11
9	23
5	9
22	51
3	14
0	0
2	5
9	18
2	11
4	19
2	5
8	27
0	5
3	5
2	8
4	14
0	0
3	7
0	5
2	13
0	10
8	31
2	13
5	10
0	3
14	56
0	9
5	39
1	4
2	16
7	30
5	12
0	3
17	65
7	20
25	66
2	3
4	26
3	15
10	46
1	2
11	40

2	24
0	8
2	8
6	8
11	38
3	10
0	8
2	8
58	229
3	22
2	7
16	76
15	53
23	116
1	7
1	3
24	69
4	31
3	3
5	30
11	27
2	6
6	7
8	22
19	84
0	4
0	0
6	42
7	19
7	24
13	38
2	8
4	16
2	13
0	3
5	52
2	4
4	20
10	27
0	4
8	18
2	16
1	7
18	92
12	18
2	8
19	77
2	5
1	3
13	37

1	4
9	46
25	63
1	3

1	2
29	112
8	64
15	100
6	8
1	9
31	119
2	3
2	4
17	68
7	48
18	91
2	6
1	9
27	103
2	6
0	0
18	72
14	44
31	140
10	34
0	2
31	147
1	5
