# Supplementary material for: Attraction of Lutzomyia longipalpis to synthetic sex-aggregation pheromone: Effect of release rate and proximity of adjacent pheromone sources
Source: PLoS Negl Trop Dis. 2018 Dec 19;12(12):e0007007. doi: 10.1371/journal.pntd.0007007 (PMC6300254; doi:10.1371/journal.pntd.0007007)
Supplement: S2 Table — (PDF) [file pntd.0007007.s002.pdf]

S2 Table

Raw data from Experiment 2 traps set 5, 10, 20 or 30 m apart

control trap = 1 lure   test trap = 5 lures

Date	replicate	HHID	Name	Position	distance apart	Treatment
07/07/2016	1	1	Leonidia	A	5m	1 Pheromone
07/07/2016	1	1	Leonidia	B	5m	5 Pheromone
07/07/2016	1	3	Marcelo	A	5m	1 Pheromone
07/07/2016	1	3	Marcelo	B	5m	5 Pheromone
11/07/2016	2	1	Leonidia	A	5m	5 Pheromone
11/07/2016	2	1	Leonidia	B	5m	1 Pheromone
11/07/2016	2	3	Marcelo	A	5m	5 Pheromone
11/07/2016	2	3	Marcelo	B	5m	1 Pheromone
12/07/2016	3	1	Leonidia	A	5m	1 Pheromone
12/07/2016	3	1	Leonidia	B	5m	5 Pheromone
12/07/2016	3	3	Marcelo	A	5m	1 Pheromone
12/07/2016	3	3	Marcelo	B	5m	5 Pheromone
13/07/2016	4	1	Leonidia	A	5m	5 Pheromone
13/07/2016	4	1	Leonidia	B	5m	1 Pheromone
13/07/2016	4	3	Marcelo	A	5m	5 Pheromone
13/07/2016	4	3	Marcelo	B	5m	1 Pheromone
14/07/2016	5	1	Leonidia	A	5m	1 Pheromone
14/07/2016	5	1	Leonidia	B	5m	5 Pheromone
14/07/2016	5	3	Marcelo	A	5m	1 Pheromone
14/07/2016	5	3	Marcelo	B	5m	5 Pheromone
15/07/2016	6	1	Leonidia	A	5m	5 Pheromone
15/07/2016	6	1	Leonidia	B	5m	1 Pheromone
15/07/2016	6	3	Marcelo	A	5m	5 Pheromone
15/07/2016	6	3	Marcelo	B	5m	1 Pheromone
18/07/2016	1	2	Arlan	A	10m	1 Pheromone
18/07/2016	1	2	Arlan	B	10m	5 Pheromone
18/07/2016	1	4	Carlos	A	10m	1 Pheromone
18/07/2016	1	4	Carlos	B	10m	5 Pheromone
19/07/2016	2	2	Arlan	A	10m	5 Pheromone
19/07/2016	2	2	Arlan	B	10m	1 Pheromone
19/07/2016	2	4	Carlos	A	10m	5 Pheromone
19/07/2016	2	4	Carlos	B	10m	1 Pheromone
20/07/2016	3	2	Arlan	A	10m	1 Pheromone
20/07/2016	3	2	Arlan	B	10m	5 Pheromone
20/07/2016	3	4	Carlos	A	10m	1 Pheromone
20/07/2016	3	4	Carlos	B	10m	5 Pheromone
21/07/2016	4	2	Arlan	A	10m	5 Pheromone
21/07/2016	4	2	Arlan	B	10m	1 Pheromone

21/07/2016	4	4	Carlos	A	10m	5 Pheromone
21/07/2016	4	4	Carlos	B	10m	1 Pheromone
25/07/2016	5	2	Arlan	A	10m	1 Pheromone
25/07/2016	5	2	Arlan	B	10m	5 Pheromone
25/07/2016	5	4	Carlos	A	10m	1 Pheromone
25/07/2016	5	4	Carlos	B	10m	5 Pheromone
26/07/2016	6	2	Arlan	A	10m	5 Pheromone
26/07/2016	6	2	Arlan	B	10m	1 Pheromone
26/07/2016	6	4	Carlos	A	10m	5 Pheromone
26/07/2016	6	4	Carlos	B	10m	1 Pheromone
18/07/2016	1	1	Leonidia	A	20m	1 Pheromone
18/07/2016	1	1	Leonidia	B	20m	5 Pheromone
18/07/2016	1	3	Marcelo	A	20m	1 Pheromone
18/07/2016	1	3	Marcelo	B	20m	5 Pheromone
19/07/2016	2	1	Leonidia	A	20m	5 Pheromone
19/07/2016	2	1	Leonidia	B	20m	1 Pheromone
19/07/2016	2	3	Marcelo	A	20m	5 Pheromone
19/07/2016	2	3	Marcelo	B	20m	1 Pheromone
20/07/2016	3	1	Leonidia	A	20m	1 Pheromone
20/07/2016	3	1	Leonidia	B	20m	5 Pheromone
20/07/2016	3	3	Marcelo	A	20m	1 Pheromone
20/07/2016	3	3	Marcelo	B	20m	5 Pheromone
21/07/2016	4	1	Leonidia	A	20m	5 Pheromone
21/07/2016	4	1	Leonidia	B	20m	1 Pheromone
21/07/2016	4	3	Marcelo	A	20m	5 Pheromone
21/07/2016	4	3	Marcelo	B	20m	1 Pheromone
25/07/2016	5	1	Leonidia	A	20m	1 Pheromone
25/07/2016	5	1	Leonidia	B	20m	5 Pheromone
25/07/2016	5	3	Marcelo	A	20m	1 Pheromone
25/07/2016	5	3	Marcelo	B	20m	5 Pheromone
26/07/2016	6	1	Leonidia	A	20m	5 Pheromone
26/07/2016	6	1	Leonidia	B	20m	1 Pheromone
26/07/2016	6	3	Marcelo	A	20m	5 Pheromone
26/07/2016	6	3	Marcelo	B	20m	1 Pheromone
05/09/2016	1	3	Marcelo	A	30m	1 Pheromone
05/09/2016	1	3	Marcelo	B	30m	5 Pheromone
05/09/2016	1	2	Arlan	A	30m	1 Pheromone
05/09/2016	1	2	Arlan	B	30m	5 Pheromone
06/09/2016	2	3	Marcelo	A	30m	5 Pheromone
06/09/2016	2	3	Marcelo	B	30m	1 Pheromone
06/09/2016	2	2	Arlan	A	30m	5 Pheromone
06/09/2016	2	2	Arlan	B	30m	1 Pheromone
07/09/2016	3	3	Marcelo	A	30m	1 Pheromone
07/09/2016	3	3	Marcelo	B	30m	5 Pheromone
07/09/2016	3	2	Arlan	A	30m	1 Pheromone
07/09/2016	3	2	Arlan	B	30m	5 Pheromone

08/09/2016	4	3	Marcelo	A	30m	5 Pheromone
08/09/2016	4	3	Marcelo	B	30m	1 Pheromone
08/09/2016	4	2	Arlan	A	30m	1 Pheromone
08/09/2016	4	2	Arlan	B	30m	5 Pheromone
12/09/2016	5	3	Marcelo	A	30m	1 Pheromone
12/09/2016	5	3	Marcelo	B	30m	5 Pheromone
12/09/2016	5	2	Arlan	A	30m	5 Pheromone
12/09/2016	5	2	Arlan	B	30m	1 Pheromone
13/09/2016	6	3	Marcelo	A	30m	5 Pheromone
13/09/2016	6	3	Marcelo	B	30m	1 Pheromone
13/09/2016	6	2	Arlan	A	30m	5 Pheromone
13/09/2016	6	2	Arlan	B	30m	1 Pheromone

Males	Females	Total
7	2	9
26	11	37
3	1	4
24	5	29
8	7	15
0	1	1
11	2	13
4	1	5
2	2	4
7	1	8
1	2	3
5	5	10
20	5	25
0	1	1
5	2	7
3	0	3
4	3	7
36	7	43
1	0	1
6	0	6
17	9	26
7	2	9
3	3	6
3	0	3
6	4	10
13	1	14
13	3	16
59	15	74
13	12	25
1	0	1
38	5	43
21	5	26
3	5	8
8	2	10
5	0	5
39	14	53
37	13	50
7	3	10

31	6	37
12	1	13
1	0	1
16	2	18
4	1	5
50	11	61
15	3	18
0	1	1
33	12	45
24	8	32
13	5	18
45	18	63
1	0	1
50	10	60
44	8	52
6	4	10
1	2	3
22	1	23
9	5	14
31	18	49
1	0	1
18	4	22
39	9	48
11	2	13
7	1	8
9	0	9
3	1	4
21	2	23
2	1	3
8	1	9
17	7	24
1	0	1
5	0	5
7	4	11
3	0	3
42	14	56
9	0	9
34	5	39
3	1	4
14	2	16
23	7	30
7	5	12
3	0	3
48	17	65
13	7	20
41	25	66

1	2	3
22	4	26
12	3	15
36	10	46
1	1	2
29	11	40
22	2	24
8	0	8
6	2	8
2	6	8
27	11	38
7	3	10
