# Supplementary material for: Attraction of Lutzomyia longipalpis to synthetic sex-aggregation pheromone: Effect of release rate and proximity of adjacent pheromone sources
Source: PLoS Negl Trop Dis. 2018 Dec 19;12(12):e0007007. doi: 10.1371/journal.pntd.0007007 (PMC6300254; doi:10.1371/journal.pntd.0007007)
Supplement: S3 Table — β coefficients convergence. For each coefficient the mean and variance are reported for the last three MCMC sub-chains (e.g. from 70,001 to 80,000; from 80,001 to 90,000; and from 90,001 to 100,000). Diff (%) is the proportion of variation in the mean and variance compared to the mean of the means and the mean of the variances. Inter, is the intercept; test is the variable containing test and controls (0 for controls and 1 for tests); ch is the interaction between test and house; cd is the interaction between test and distance; h is the house (house number 2, 3 and 4); and d is the distance (10m, 20m and 30m). (PDF) [file pntd.0007007.s003.pdf]

S3 Table. Experiment 2. β coefficients convergence. For each coefficient the mean and variance are reported for the last three MCMC sub-chains (e.g. from 70,001 to 80,000; from 80,001 to 90,000; and from 90,001 to 100,000). Diff (%) is the proportion of variation in the mean and variance compared to the mean of the means and the mean of the variances. Inter, is the intercept; test is the variable containing test and controls (0 for controls and 1 for tests); ch is the interaction between test and house; cd is the interaction between test and distance; h is the house (house number 2, 3 and 4); and d is the distance (10m, 20m and 30m).

Coeff.	Mean1	Mean2	Mean3	Diff(%)		Var1	Var2	Var3	Diff(%)
**Inter**	3.355	3.341	3.344	0.432		0.023	0.026	0.021	20.046
**test**	1.702	1.722	1.709	1.131		0.027	0.031	0.027	14.866
**h2**	-0.087	-0.070	-0.073	21.229		0.060	0.051	0.054	16.606
**h3**	-0.322	-0.302	-0.318	6.363		0.024	0.023	0.024	1.094
**h4**	1.071	1.078	1.076	0.656		0.093	0.085	0.096	12.495
**d10**	0.137	0.136	0.152	10.770		0.083	0.084	0.089	6.420
**d20**	0.767	0.780	0.775	1.679		0.026	0.029	0.030	12.351
**d30**	1.023	1.019	1.026	0.651		0.042	0.043	0.048	12.026
**ch2**	-0.451	-0.442	-0.458	3.558		0.080	0.064	0.070	21.394
**ch3**	-0.529	-0.543	-0.529	2.675		0.032	0.030	0.033	11.937
**ch4**	-0.758	-0.742	-0.775	4.339		0.127	0.111	0.120	13.029
**cd10**	0.234	0.208	0.226	11.944		0.106	0.108	0.114	6.966
**cd20**	-0.279	-0.296	-0.291	5.913		0.033	0.036	0.036	8.301
**cd30**	-0.049	-0.076	-0.061	44.959		0.056	0.057	0.062	10.087
