# Supplementary material for: Attraction of Lutzomyia longipalpis to synthetic sex-aggregation pheromone: Effect of release rate and proximity of adjacent pheromone sources
Source: PLoS Negl Trop Dis. 2018 Dec 19;12(12):e0007007. doi: 10.1371/journal.pntd.0007007 (PMC6300254; doi:10.1371/journal.pntd.0007007)
Supplement: S4 Table — Male (A) and Female (B) response to different numbers of lures. (PDF) [file pntd.0007007.s004.pdf]

S4 Table a (male)

Coeff.	Mean	Variance	CR.0.05	CR.0.95
ßl2	3.628698	0.026954	3.359849	3.899967
ßl5	3.915633	0.020552	3.67941	4.152306
ßl10	4.265358	0.01408	4.069575	4.46264
ßl20	3.421459	0.033824	3.121494	3.730014
ßl50	4.357963	0.012636	4.171984	4.542898
ßcl2	0.599803	0.041827	0.257819	0.932112
ßcl5	1.132483	0.026987	0.863323	1.399595
ßcl10	1.313775	0.018149	1.090374	1.534425
ßcl20	1.781539	0.039519	1.451616	2.109916
ßcl50	1.588366	0.015314	1.383703	1.793616

S4 Table b (female)

Coeff.	Mean	Variance	CR.0.05	CR.0.95
ßl2	3.04932	0.048404	2.690802	3.411015
ßl5	2.717343	0.066092	2.293151	3.139511
ßl10	3.314842	0.037037	3.00102	3.632147
ßl20	2.037754	0.125539	1.455286	2.632318
ßl50	2.954296	0.052863	2.578414	3.326654
ßcl2	0.284829	0.082582	-0.19253	0.758526
ßcl5	1.255314	0.086167	0.775174	1.743136
ßcl10	1.107696	0.04946	0.742806	1.471249
ßcl20	2.204682	0.139845	1.590475	2.821327
ßcl50	1.807886	0.061213	1.399844	2.211458
