# Supplementary material for: Attraction of Lutzomyia longipalpis to synthetic sex-aggregation pheromone: Effect of release rate and proximity of adjacent pheromone sources
Source: PLoS Negl Trop Dis. 2018 Dec 19;12(12):e0007007. doi: 10.1371/journal.pntd.0007007 (PMC6300254; doi:10.1371/journal.pntd.0007007)
Supplement: S3 Fig — β coefficients histograms from the posterior distributions. β: inter is the model intercept; test is the variable containing test and controls (0 for controls and 1 for tests); ch is the interaction between test and house; cl is the interaction between test and pheromones; h is referred to house (house number 2, 3 and 4); and l is referred to pheromone lures (2, for 5 lures; 3 for 10 lures; 4 for 20 lures; and 5 for 50 lures). (PDF) [file pntd.0007007.s007.pdf]

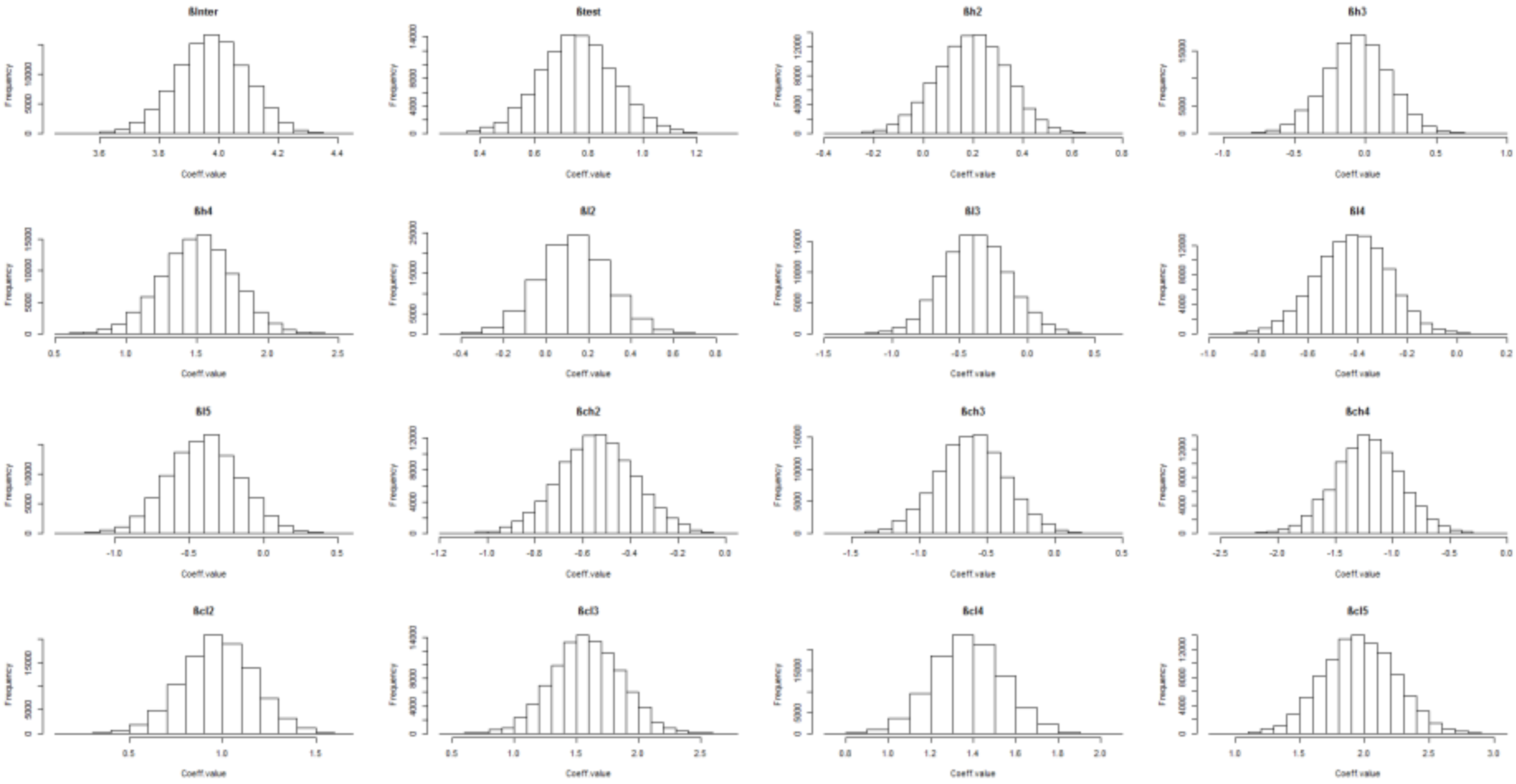

S3 Fig Experiment 1. *β* coefficients histograms from the posterior distributions. *β*: **inter** is the model intercept; **test** is the variable containing test and controls (0 for controls and 1 for tests); **ch** is the interaction between test and house; **cl** is the interaction between test and pheromones; **h** is referred to house (house number 2, 3 and 4); and **l** is referred to pheromone lures (2, for 5 lures; 3 for 10 lures; 4 for 20 lures; and 5 for 50 lures).
