# Supplementary material for: Attraction of Lutzomyia longipalpis to synthetic sex-aggregation pheromone: Effect of release rate and proximity of adjacent pheromone sources
Source: PLoS Negl Trop Dis. 2018 Dec 19;12(12):e0007007. doi: 10.1371/journal.pntd.0007007 (PMC6300254; doi:10.1371/journal.pntd.0007007)
Supplement: S4 Fig — β coefficients histograms from the posterior distributions. Explanation of titles after the β: inter, is the intercept of the model; test is the variable containing test and controls (0 for controls and 1 for tests); ch is the interaction between test and house; cd is the interaction between test and distance; h is house (house number 2, 3 and 4); and d refers to distances (10m, 20m, 30m). (PDF) [file pntd.0007007.s008.pdf]

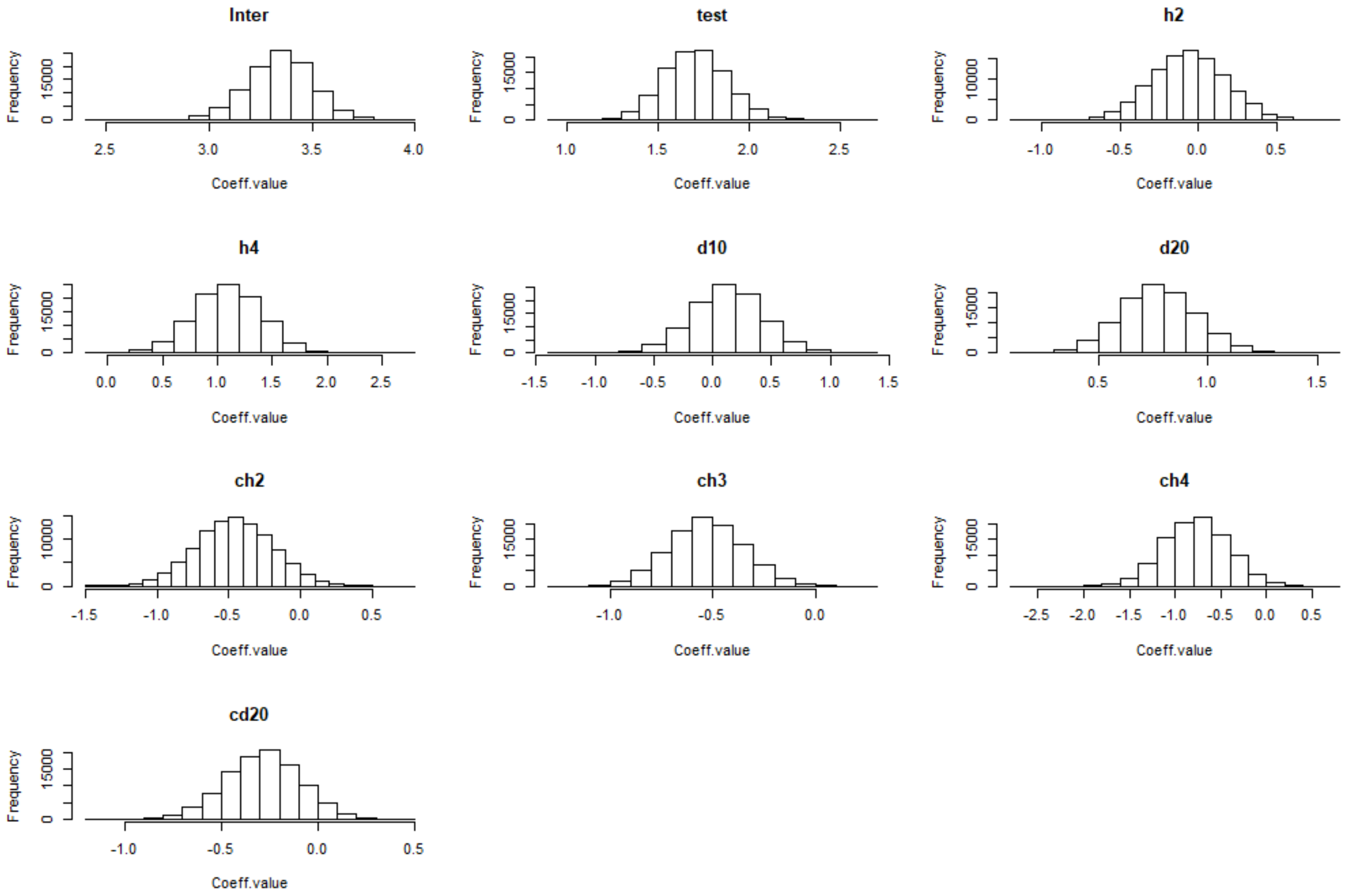

S4 Figure. Experiment 2. β coefficients histograms from the posterior distributions. Explanation of titles after the β: **inter**, is the intercept of the model; **test** is the variable containing test and controls (0 for controls and 1 for tests); **ch** is the interaction between test and house; **cd** is the interaction between test and distance; **h** is house (house number 2, 3 and 4); and **d** refers to distances (10m, 20m, 30m).
