# Supplementary material for: Attraction of Lutzomyia longipalpis to synthetic sex-aggregation pheromone: Effect of release rate and proximity of adjacent pheromone sources
Source: PLoS Negl Trop Dis. 2018 Dec 19;12(12):e0007007. doi: 10.1371/journal.pntd.0007007 (PMC6300254; doi:10.1371/journal.pntd.0007007)
Supplement: S5 Fig — β coefficients histograms from the posterior distributions of the number of pheromone lures and their interaction with test/control. Explanation of titles: l is the number of pheromone lures (1 is 2 lures; 2 is 5 lures; 3 is 10 lures; 4 is 20 ures; and 5 is 50 lures); cl is the interaction between test and pheromone. (PDF) [file pntd.0007007.s009.pdf]

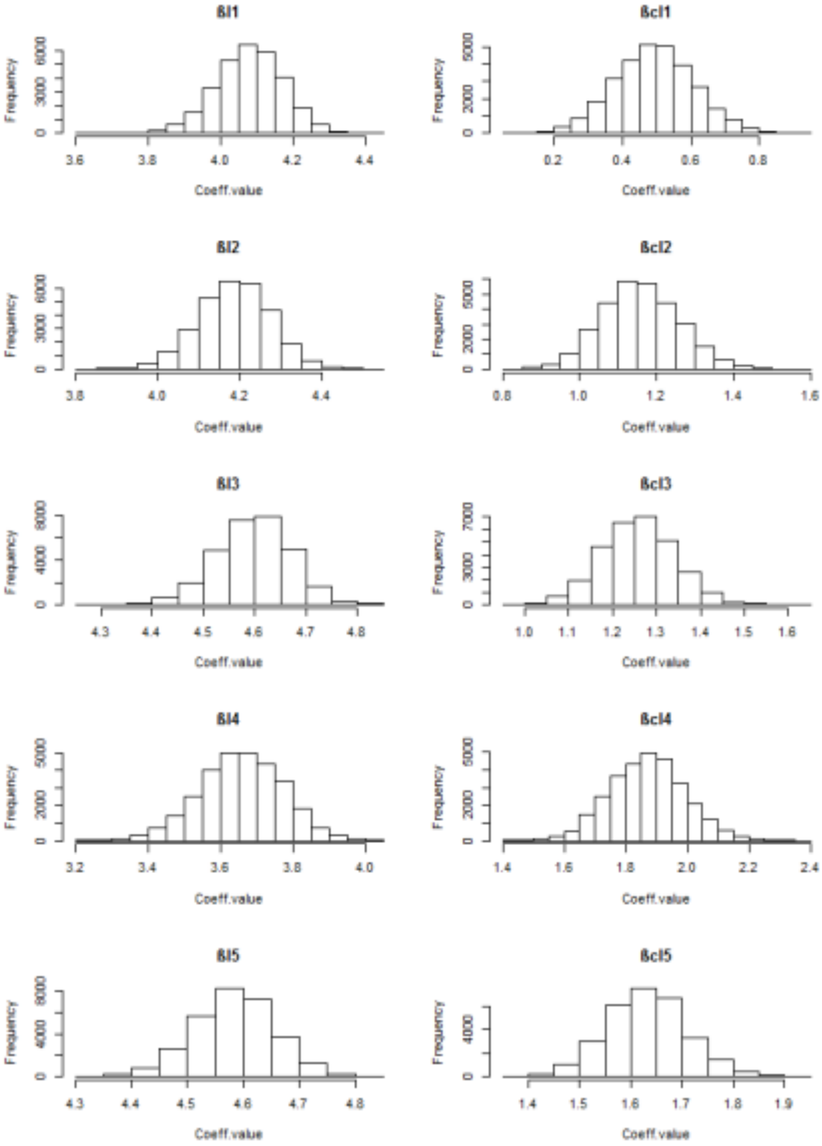

S5 Fig Experiment 1. β coefficients histograms from the posterior distributions of the number of pheromone lures and their interaction with test/control. Explanation of titles: l is the number of pheromone lures (1 is 2 lures; 2 is 5 lures; 3 is 10 lures; 4 is 20 lures; and 5 is 50 lures); cl is the interaction between test and pheromone.
